# BTA Deep Hole Vibration Drilling for Nickel-Based Alloys: Cooling Patterns and Cutter Tooth Wear Mechanisms

**DOI:** 10.3390/ma15228178

**Published:** 2022-11-17

**Authors:** Yuhua Shi, Jianming Zheng, Pei Feng, Peng Shang, Chi Liu, Ting Chen, Shijie Shan

**Affiliations:** 1 School of Mechanical and Precision Instrument Engineering, Xi’an University of Technology, Xi’an 710048, China; 2 School of Equipment Management and Support, Engineering University of PAP, Xi’an 710078, China; 3 Science and Technology on Plasma Dynamics Lab, Air Force Engineering University, Xi’an 710038, China

**Keywords:** nickel-based alloys, BTA drilling, vibration drilling, drilling temperature, cutter tooth wear

## Abstract

The high cutting temperature and poor thermal diffusion efficiency of nickel-based alloys during deep hole machining have become technical challenges in the hole machining field. In this paper, a finite element simulation model of Inconel-718 BTA ordinary drilling and vibration drilling processes was established by using Deform-3D finite element simulation software. The variations in the temperatures of the tool teeth and the workpiece at different positions of the nickel-based alloy under ordinary drilling and vibration drilling were investigated. Additionally, the wear pattern of each tool tooth under the two drilling methods was further analyzed by building an experimental platform for workpiece temperature detection, which reveals the wear and cooling mechanism of nickel-based alloy BTA deep hole drilling. The results show that the average temperatures of the external, intermediate, and central teeth were reduced by 18.1%, 21.1%, and 17.8%, respectively, during vibration drilling. In addition, the workpiece hole wall and hole bottom temperatures were reduced by 5.7% and 4.6%, respectively. To conclude, the experimental tests were consistent with the simulated temperature trends. BTA vibration drilling optimizes the heat exchange conditions between the cutter teeth and the workpiece during the drilling of nickel-based alloys, which effectively reduces the cutting temperature and, thus, improves the wear resistance of the cutter teeth.

## 1. Introduction

Nickel-based alloys are widely used in the manufacture of aero-engine components, such as combustion chambers, guiding devices, turbine blades, and turbine disks due, to their high strength, high corrosion resistance, and excellent thermal stability and thermal fatigue under high temperatures. They are currently the most popular, largest and most widely used class of alloys for aero engines [1,2]. However, traditional cutting methods for hole machining of nickel-based alloys produce high cutting forces, high cutting temperatures, and serious tool wear problems [3,4]. In addition, nickel-based alloy parts in deep hole machining face the challenges of discharge chips and cutting heat dissipation efficiency, which seriously affect the tool life and the quality of surface machining of parts. Consequently, deep hole machining of nickel-based alloys is currently a technical problem in the hole-making process of difficult-to-machine materials [5,6,7,8].

At present, domestic and foreign scholars have conducted some research on the factors influencing the cutting temperature during the cutting of nickel-based alloys. Marusich [9], Coelho [10], Liu [11], and Arrazola [12] et al. found that in ordinary cutting, cutting parameters, cutter teeth geometry, and tool materials are all important factors affecting the cutting temperature of nickel-based alloys, tool wear, and surface finish quality. 

However, in the process of cutting nickel-based alloys with the ordinary cutting method, it is easy to produce high-temperature deformation and breakage, and the tool wear is intensified, which will affect the machining quality of the workpiece surface. Therefore, it is urgent to optimize the cutting method to reduce the surface cutting temperature and improve the machining quality [13]. 

Vibratory cutting is an improved method for reducing cutting temperatures in machining. Its intermittent cutting process allows the area of interaction between the tool and the workpiece to be disconnected periodically. During the non-cutting phase, the entry of the cutting fluid into the cutting zone enhances the cooling rate, thus hindering tool wear and improving surface quality [14,15,16]. Stephenson [17], Masahiko [18], and Jiang [19], et al. established a theoretical model of the transient cutting temperature of the tool during interrupted cutting. They found that reducing the ratio of cutting time to non-cutting time can reduce the tool’s cutting temperature, and make the relatively gentle change in tool temperature during the cutting cycle. It shows that interrupted cutting is an effective means to reduce cutting temperature. Azghandi [20] and Sadek [21] et al. studied the influence law of cutting parameters, such as speed, feed rate, and frequency, on the hole quality of machining composite materials during vibration drilling. The results showed that the optimized cutting parameters could reduce the cutting temperature by 50%. Pecat [22], Xu [23], and Hussei [24] et al. found that low-frequency vibration drilling can produce interrupted chips, reduce mechanical damage, and lower the cutting temperature through an experimental study of the low-frequency vibration drilling process of CFRP/Ti6Al4V stacked material. Kong Fanxia et al. [25] demonstrated that vibration drilling shows a great cooling effect, which can effectively improve tool wear resistance via dry drilling of nickel-based high-temperature alloys with twist drills. However, these studies mainly analyzed the temperature at one point of the cutting edge of the tool teeth, which has some error and makes it difficult to comprehensively analyze the overall temperature field distribution of the tool teeth. At present, domestic and foreign scholars have reported the vibration drilling of different materials and the cutting temperature in the process of vibration drilling. However, the processing method that has mainly been focused on is twist drilling, and the processing materials have mainly been composite materials. However, nickel-based alloys have higher hardness and greater cutting forces than composites, resulting in higher cutting temperatures. At high temperatures, it is easy for the workpiece to react with the coating and base material of the tool, which will aggravate the tool wear.

BTA deep hole drilling is an inner-chip removal self-guiding deep hole processing technology, for which chip size and shape will have a direct impact on drilling temperature and hole processing quality. At the same time, vibration drilling makes the drilling into a dynamic and pulse cutting process due to the additional vibration, thus producing a series of excellent technological results. Therefore, based on the heat transfer mechanism of BTA deep hole vibration drilling, the temperature field of the cutter teeth and temperature variation of the workpiece at different positions with time of the nickel-based alloy under ordinary drilling and vibration drilling conditions were investigated. By comparing the wear patterns of each cutter tooth under the two drilling methods, the wear and temperature reduction mechanism of BTA deep hole vibration drilling of nickel-based alloys was revealed. This work provides a new way to improve the thermal conductivity of nickel-based alloy deep hole machining, reduce the cutting temperature, and improve tool wear. This paper is of great significance to improve the quality of nickel-based alloy machining and extend tool life.

## 2. Analysis of the Heat Transfer Mechanism of BTA Deep Hole Drilling

### 2.1. Generation and Transmission of Drilling Heat

**Figure 1:**
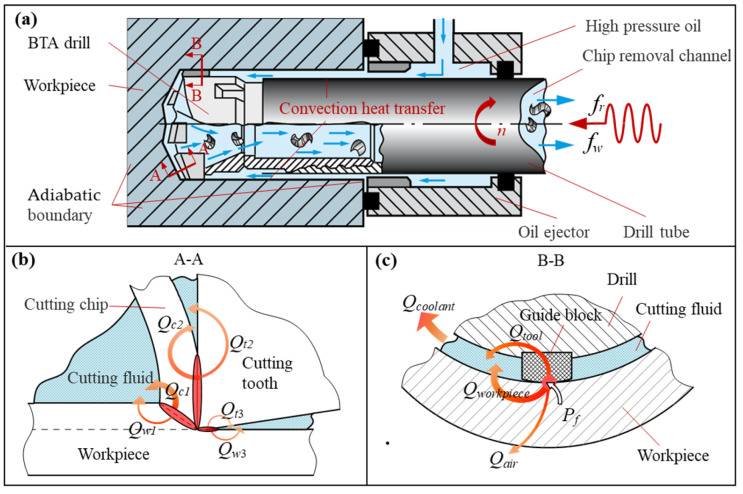
(**a**) The working principle diagram and heat conduction boundary conditions of the BTA deep hole vibration drilling. (**b**) Heat conduction model of the cutter teeth. (**c**) Guide block extrusion friction heat conduction model.

As shown in Figure 1a, a tool-rotating internal chip discharge self-guided deep hole drilling system was used in this experiment. Firstly, the workpiece is fixed to the table while the staggered tooth BTA drills is mounted on the hollow drill tube. Secondly, the drill tube is moved in a main rotational motion at a constant speed *n*. The feed motor drives the drill tube in an axial feeding motion at a constant feed *f_r_*, while the vibration motor drives the drill tube in an amplitude *a* and vibration frequency *f_w_* along the axial low-frequency mechanical vibration. The heat transfer process between the tool system and the workpiece during the drilling process is transformed into the conduction of heat in a semi-infinite medium under the action of a heat source and convective heat transfer boundary conditions, as shown in Figure 1. In ordinary drilling, heat transfer is simplified to a steady-state temperature field acting on constant heat source intensity. In vibration drilling, the cutting forces vary periodically, and the heat transfer is regarded as a transient temperature field under alternating heat source intensity.

As a composite machining method where the cutter teeth cut first and then the guide block is extruded, the heat of BTA drilling comes from two main sources. The first heat source comes from the cutting heat of the cutter teeth. This heat source consists of the work done by the shear deformation of the material in the first deformation zone, the heat generated by the friction between the rake face of the cutter teeth and the chip in the second deformation zone, and the friction between the flank face of the cutter tooth and the machined surface in the third deformation zone. This heat is continuously diffused into the workpiece (*Q_w_*_1_, *Q_w_*_3_), cutter teeth (*Q_t_*_2_, *Q_t_*_3_), and chip (*Q_c_*_1_, *Q_c_*_2_) via thermal conduction, as shown in Figure 1b. The second heat source is from the extrusion friction heat source formed by the work done by friction between the external tooth sizing edge in the first guide block and the second guide block and the hole wall (*P_Fd_, P_f_*_1_*, P_f_*_2_), as shown in Figure 1c. This heat enters the guide block (*Q_tool_*) and the workpiece (*Q_workpiece_*) through thermal conduction and continuously diffuses to the cutting fluid (*Q_coolant_*) and the outside of the workpiece (*Q_air_*). In addition, the inner and outer surface of the drills and the surface of the machined hole wall are set as the convective heat transfer boundaries. The outer surface of the workpiece is set as an adiabatic boundary.

### 2.2. Effect of Axial Vibration on Heat Transfer Processes

**Figure 2:**
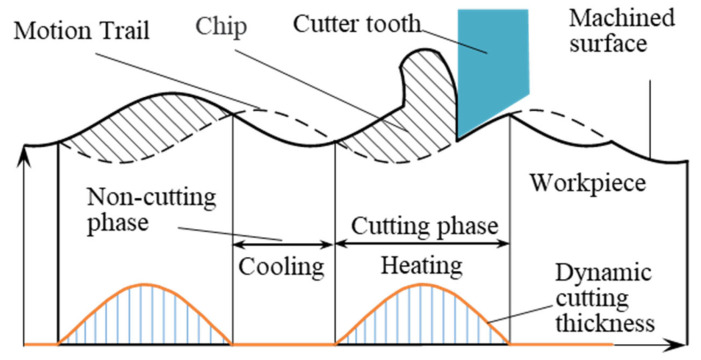
Schematic diagram of the vibratory cutting process.

Figure 2 shows a schematic diagram of the vibratory cutting process. During vibratory cutting, the cutter teeth periodically cut into and out of the workpiece. The tool temperature rise process, therefore, consists of two phases, heating and cooling, which constitute an intermittent heat transfer model. The axial transient cutting thickness of vibration drilling is no longer constant but varies periodically with the angle of rotation of the drill. When the drilling parameters meet certain conditions, the drilling process periodically results in a non-cutting phase with a instantaneous cutting thickness of zero. At this point, the tool is completely separated from the workpiece and the cutting heat source of the cutter teeth stops generating heat, while the cutting fluid is able to enter the cutting zone and cool the cutting zone through forced convection heat transfer.

Under intermittent vibration drilling conditions, each vibration cycle *T* consists of cutting time *t_c_* and non-cutting time *t_i_*. The cutting time ratio *K* is usually defined as:

(1) $$K=\frac{t_{c}}{T}$$

where *T* = 60/(*n·ω_f_*). ω*_f_* is the frequency ratio that indicates the number of axial vibrations of the drill rotating one cycle.

During the vibration drilling process, the duration of the cutting and non-cutting phase can be adjusted by changing the combination of process parameters, thus controlling the time between the heating and cooling phases of the cutter teeth.

### 2.3. Vibration Drilling Cutter Tooth Heat Transfer Model

**Figure 3:**
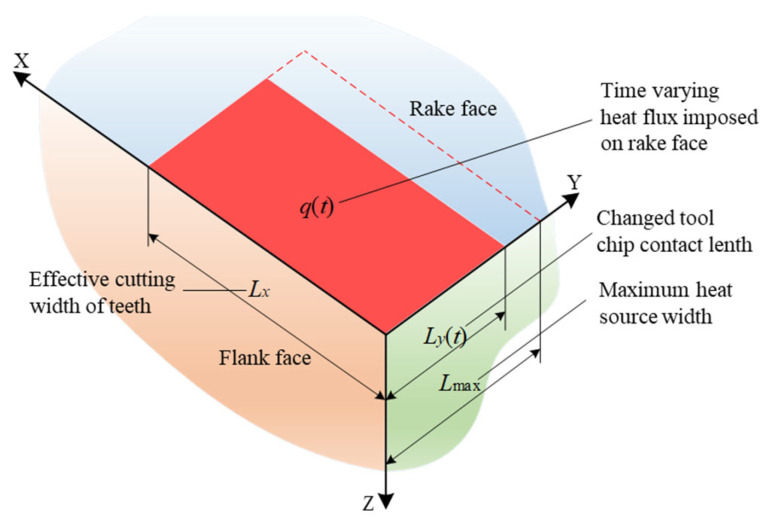
Geometric model of heat conduction on the rake face of cutter teeth in vibration drilling.

Neglecting the effect of the corner radius on the temperature field distribution of the cutter teeth, the cutter teeth are simplified to a semi-infinite three-dimensional space, as shown in Figure 3. The red rectangular surface on the rake face represents the uniform heat source applied on the rake face. The flank face and the sides of the teeth can be considered adiabatic surfaces, while the initial temperature is assumed to be zero at infinity throughout the semi-infinite space.

During vibration drilling, the tool-chip contact length varies periodically with the drilling time and can be calculated by the following equation [26,27]:

(2) $$L_{y}\left(t\right)=l_{c}\left(t\right)=h\left(t\right)\frac{k_{w}+2}{2}\frac{\sin\left(\varphi +\beta -\gamma_{act}\right)}{\sin\varphi \cos\beta }$$

where *h(t)* is dynamic cutting thickness, *k_w_* stands for the pressure distribution index on the tool–chip interface, *φ* is the shear angle, *β* is the friction angle, and *γ_act_* is the normal rake angle of the tooth.

Neglecting the effects of thermal radiation and cutting fluid convective heat transfer on the temperature field, the control equations and boundary conditions for the heat transfer model of the rake face of the cutter teeth in vibration drilling can be expressed as Equations (3) and (4), respectively.

(3) $$\frac{\partial^{2}T_{em}}{\partial x^{2}}+\frac{\partial^{2}T_{em}}{\partial y^{2}}+\frac{\partial^{2}T_{em}}{\partial z^{2}}=\frac{1}{a_{t}}\frac{\partial T_{em}}{\partial t}$$

(4) $$-\lambda \frac{\partial T_{em}}{\partial z}=q\left(t\right),z=0;0\leq x\leq L_{x},0\leq y\leq L_{y}\left(t\right)$$

where *T_em_* is the temperature at a point on the rake face of cutter teeth, *a_t_* is the thermal diffusivity of the cutter tooth material, and *λ* is the coefficient of heat conduction.

For any point (*x*,*y*,*z*) on the tool–chip interface of the rake face, the temperature *T_em_*(*x*,*y*,*z,t_e_*) at time *t_e_* can be calculated by Green’s function [28,29] and the relevant equation is as follows:

(5) $$T_{em}\left(x,y,z,t_{e}\right)=\frac{a_{t}}{\lambda }\int_{0}^{t_{e}}\int_{0}^{L_{x}}\int_{0}^{L_{y}\left(t\right)}\theta_{G}\left(x,y,z,x_{p},y_{p},0,D\right)q\left(t\right)dy_{p}dx_{p}dt$$

where *θ_G_* is the temperature distribution in a semi-infinite space after the action time, *t* seconds, of the instantaneous point heat source *(x_p_*, *y_p_*, 0*)* expressed by Green’s function.

In the plane 0 ≤ *x* ≤ *L_x_*, 0 ≤ *y* ≤ *L_y_(t)*, the equation of Green’s function for an instantaneous point heat source is:

(6) $$\theta_{GR}\left(x,y,z,L_{x},L_{y\left(t\right)},D\right)=\frac{1}{2\sqrt{\pi }D}exp\left(\frac{-z^{2}}{D^{2}}\right)\theta_{GU}\left(x,L_{x},D\right)\theta_{GU}\left(y,L_{y}\left(t\right),D\right)$$

where *θ_GU_* and *D* can be calculated by the following equations:

(7) $$\theta_{GU}\left(u,L,D\right)=erf\left(\frac{L+u}{D}\right)+erf\left(\frac{L-u}{D}\right)$$

(8) $$D=2\sqrt{a_{t}\left(t_{e}-t\right)}$$

Finally, the temperature *T(x*,*y*,*z,t_e_)* at any point (*x*,*y*,*z*) on the tool–chip interface of the rake face can be expressed as:

(9) $$T_{em}\left(x,y,z,t_{e}\right)=\frac{a_{t}}{\lambda }\int_{0}^{t_{e}}\theta_{GR}q\left(t\right)dt$$

During ordinary drilling, the actual heat *Q* is transferred to the cutter teeth per unit time and the tool–chip contact area *A_c_* are both kept constant, so the heat flux *q* also is kept constant. During vibration drilling, the cutting force and tool–chip contact length both vary periodically, resulting in a periodic variation in the actual heat *Q* transferred to the cutter teeth per unit time and chip contact area *A_c_*. Consequently, the heat flux *q* also varies periodically with the drilling time, and the temperature of the rake face also varies periodically.

## 3. Finite Element Simulation Modeling and Experimental Scheme

### 3.1. Finite Element Modeling

#### 3.1.1. Geometric Modeling

**Figure 4:**
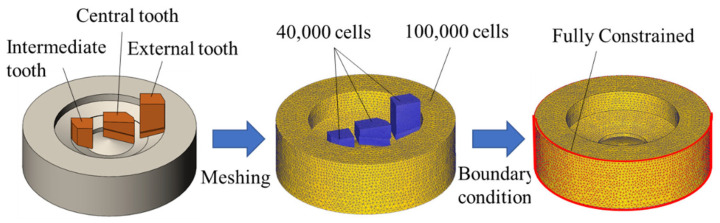
Finite element model of drilling.

In BTA deep hole drilling, it is assumed that after cutting by the cutter teeth, the hole wall is extruded by the external tooth sizing edge. Hence, the Φ17.75 staggered-tooth BTA drills were simplified into three cutter teeth, including the intermediate tooth, central tooth, and external tooth, for drilling simulation analysis, as shown in Figure 4.

The tool is set up as a rigid body, and the material is WC carbide. The workpiece is set up as a plastic body, and the material is Inconel 718. The characteristic parameters of two materials are shown in Table 1.

#### 3.1.2. Mesh Generation

In the Deform-3D preprocessor, the intermediate tooth, central tooth, external tooth, and the workpiece are meshed separately. To prevent mesh distortion during the simulation and to increase the efficiency of the calculation, an absolute cell type and adaptive meshing (AMG) were used. As shown in Figure 4, the minimum mesh cell edge length of the workpiece was set to 0.2 times the feed to produce regular chips during the drilling process.

#### 3.1.3. Material Constitutive Models and Failure Criteria

The Johnson–Cook flow stress model was used to represent the constitutive relation of Inconel 718 with the following expressions.

(10) $$\sigma =\left(A+B\varepsilon^{n}\right)\left[1+C\;ln\left(\frac{\dot{\varepsilon }}{{\dot{\varepsilon }}_{0}}\right)\right]\left[1-{\left(\frac{T-T_{r}}{T_{m}-T_{r}}\right)}^{m}\right]$$

where ${\dot{\varepsilon }}_{0}$ is reference strain rate; $\dot{\varepsilon }$ is strain rate; σ is equivalent flow stress; *T_r_* is reference temperature (20 °C); *T_m_* is the melting temperature of the material (1340 °C); and *A*, *B*, *C*, *n*, and *m* are model parameters of the yield stress, strain hardening coefficient, strain rate coefficient, strain hardening index, and thermal softening index of a material at a given temperature and strain rate, respectively. Specific parameters are shown in Table 2.

The J–C shear failure criterion is used to determine whether the material has failed based on whether the equivalent plastic strain at the cell integration point reaches a predetermined value. When the failure value *D* of a grid cell reaches 1, the material is judged to have failed, and chip separation is achieved by deleting the grid cell. The expression for the failure parameter *D* is as follows:

(11) $$D=\sum \frac{\Delta \varepsilon }{\varepsilon_{f}}$$

where *ε_f_* is the failure strain and Δ*ε* is the failure strain increment.

The shear friction model is used to define the friction conditions between the tool chip and workpiece chip. The model assumes that the frictional shear stress *τ* is proportional to the workpiece material shear stress *k*. The expression is as follows.

(12) $$\tau =m_{c}k$$

where *m_c_* is the coefficient of shear friction, which was set as 0.6 in this paper.

#### 3.1.4. Simulation Control and Boundary Condition Setting

During the simulation, the simulation type was set to incremental, and the simulation method was set to deformation and heat transfer. The total simulation time was set to 5 s. Due to the large deformation in the BTA deep hole drilling simulation, the sparse solver with good convergence was used. The Newton–Raphson algorithm was chosen to improve the computational efficiency and to reduce the number of iterations as much as possible while ensuring the computational accuracy, and when the iterations did not converge, the system was automatically transferred to the direct algorithm for solution.

The initial temperature of the drill and workpiece (ambient temperature) was set at 20 °C. The effect of the cutting fluid on the temperature field was defined by setting the convective heat transfer coefficient at the boundary, and the heat transfer boundary conditions are shown in Figure 2. In this paper, the convective heat transfer coefficient at the heat transfer boundary was set to 2 N/s/mm/°C, and the convective heat transfer coefficient at the adiabatic boundary was set to 0.

The workpiece was set to full constraint (as shown in Figure 4). For ordinary drilling, the rotation speed of the three cutter teeth around the center axis and the feed speed along the negative direction of the z-axis are set separately to define the drills motion. For vibration drilling, axial vibration is applied by means of an instantaneous feed velocity function. The feed displacement *s* and feed velocity *v* along the z-axis direction with respect to time *t* are calculated as follows:

(13) $$s=asin\left(2\pi f_{w}t\right)+\frac{nf_{r}}{60}t$$

(14) $$v=2\pi af_{w}cos\left(2\pi f_{w}t\right)+nf_{r}$$

where *a* is the amplitude, *f_w_* is the vibration frequency, *n* is the spindle rotation speed, and *f_r_* is the feed per revolution.

### 3.2. Experimental

**Figure 5:**
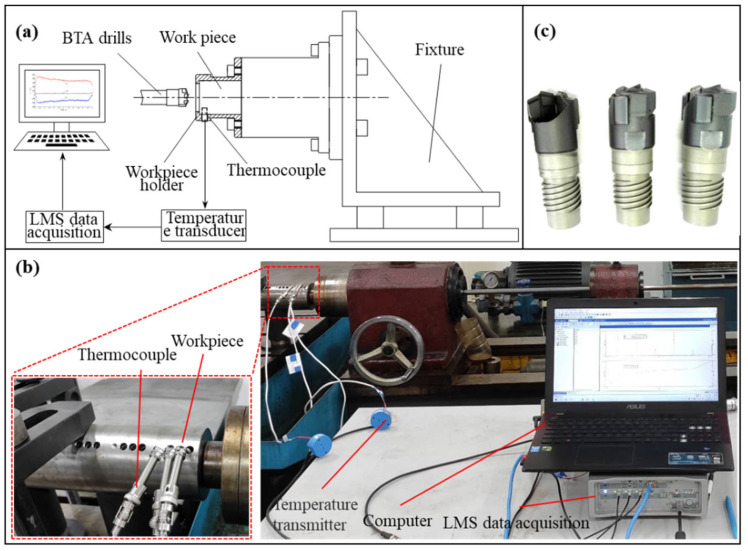
(**a**) Experimental principle of temperature measurement and detection. (**b**) Experimental facility. (**c**) Φ17.75 mm staggered-tooth BTA drills.

The experimental process used Φ17.75 mm staggered-tooth BTA drills, as shown in Figure 5c. The workpiece material was Φ80 mm nickel-based 718 bar stock. To measure the temperature change curve at specific points of the workpiece, a temperature measurement system was set up, as shown in Figure 5. At the distance of 1.5 mm from the wall of the hole to be drilled and 10 mm from the plane of the hole and at an axial position of 1.5 mm from the bottom of the hole to be machined, a small hole of Φ4 mm was pre-machined to embed an artificial thermocouple. This thermocouple was used to measure the temperature change between the wall and the bottom of the hole inside the workpiece. The thermocouple sensor (probe type K) then converted the temperature at a specific point inside the workpiece into a current or voltage signal corresponding to the temperature, which was read into the computer by the LMS data acquisition system.

## 4. Discussion

The drilling process parameters are shown in Table 3.

### 4.1. Temperature Field Analysis of Cutter Teeth during Drilling

In ordinary drilling, the tool is in continuous contact with the workpiece. The contact area of the cutter teeth and chips mainly relies on heat conduction to diffuse heat, and the cutting temperature of each tooth increases gradually with the drilling depth. When the cutting heat generated by the drilling process and the heat transferred through various media reach an equilibrium state, the temperature field of the cutter teeth tends to stabilize.

**Figure 6:**
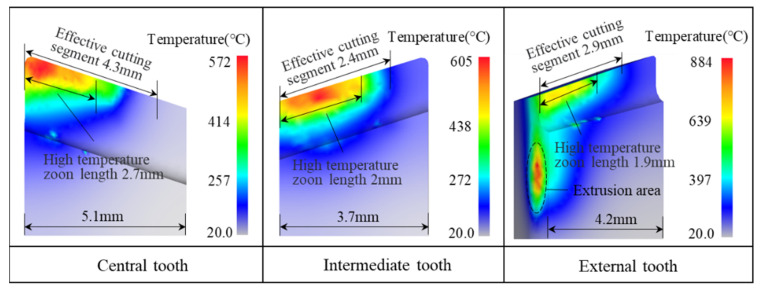
Temperature field on each cutter tooth for ordinary drilling.

Figure 6 shows a nephogram of the temperature field of each cutter tooth in the ordinary drilling thermal equilibrium state. The high-temperature area of the central tooth is close to the effective cutting section at the tip of the cutter tooth, with a maximum temperature of 572 °C. The heat in the area of the cutter teeth not involved in cutting mainly comes from the heat transfer in the cutting section. The high-temperature area of the intermediate tooth is in the middle of the effective cutting section, with a maximum temperature of 605 °C, which is 5.77% higher than the maximum temperature of the central tooth. The maximum temperature of the rake face of the external tooth is 685 °C, and the maximum temperature of the sizing edge of the external tooth is 883 °C. For the entire drills, the temperature of the rake face of the cutter tooth rises gradually as the radius increases, indicating that the cutting speed has a greater influence on the cutting heat. Therefore, a suitable speed should be selected during machining to avoid high cutting temperatures.

**Figure 7:**
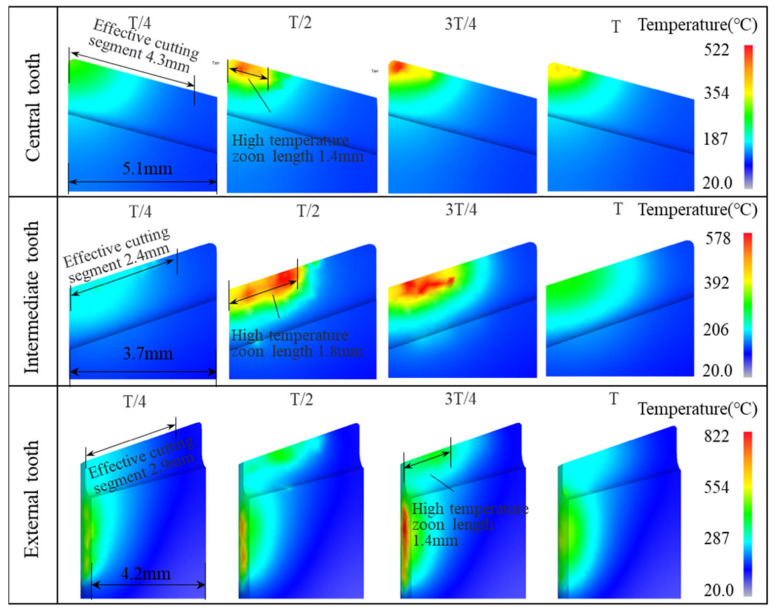
Variation temperature field of the cutter teeth for one vibration cycle of vibration drilling.

During vibration drilling, the cutter teeth temperature field varies cyclically due to the periodic disconnection of the cutting zone and cutting zone effectively cooling. Figure 7 shows the change in the temperature field of the cutter teeth for each vibration cycle of vibration drilling. The entire vibration cycle is divided into four stages. In the first stage, from the beginning to the T/4 cycle, the cutter teeth gradually approach the workpiece, which is not in full contact with the workpiece, resulting in good heat exchange conditions. At the second stage, the T/2 cycle, the tool–chip contact length reaches its maximum value in the entire cycle. The rake face and flank face of the cutter teeth are in full contact with the workpiece, and the cutting heat generated in the cutting zone is difficult to diffuse out, resulting in continuous heating of the cutter teeth. At the third stage, the 3T/4 cycles, the cutter teeth start to move away from the workpiece, the tool–chip contact length gradually decreases, and the high-temperature area of the cutter tooth decreases. At the end of a cycle, the cutter teeth are separated from the workpiece, and the heat exchange environment is good, allowing continuous heat dissipation from the cutter teeth. Similarly to ordinary drilling, the high-temperature zone of the central tooth of vibration drilling is also concentrated at the tip of the cutter tooth. However, the area of the high-temperature zone is smaller, and the length of the high-temperature zone is reduced from 2.7 mm to 1.4 mm for the central tooth, from 2 mm to 1.8 mm for the intermediate tooth, and from 1.9 mm to 1.4 mm for the external tooth. In addition, the temperature gradient in the temperature field of the rake face is larger. This indicates a significant improvement in the heat transfer conditions of the cutter teeth compared to ordinary drilling. During the whole vibration cycle, the maximum temperature on the rake face of the central tooth was 522 °C, a decrease of 8.80% compared to ordinary drilling. The maximum temperature on the rake face of the intermediate teeth was 578 °C, a decrease of 4.47% compared to ordinary drilling. The maximum temperatures on the rake face of the external teeth and the sizing edge were 638 °C and 822 °C, respectively, a decrease of 6.86% and 7.01% compared to ordinary drilling.

The maximum and average values of the temperature in the high-temperature area of each tooth for ordinary drilling and vibration drilling in thermal equilibrium were compared (as shown in Table 4). As can be seen, the cooling effect of the cutter teeth is obvious. It shows that vibration drilling can significantly improve the heat exchange conditions for cutter tooth drilling.

### 4.2. Workpiece Temperature Analysis during Drilling

**Figure 8:**
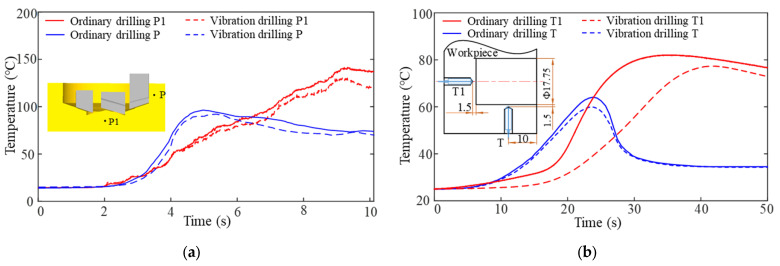
(**a**) Simulated internal temperature rise curve of workpiece by vibration drilling and ordinary drilling; (**b**) Experimental measured changes in the internal temperature of the workpiece by vibration drilling and ordinary drilling.

The temperature of the workpiece during the drilling process is directly related to the cutting heat of the tool, while the temperature variation of the workpiece has a significant impact on the heat transfer of the entire drilling system and the stability of the drilling process. Figure 8a shows the temperature rise curve inside the workpiece during vibration drilling and ordinary drilling obtained from the simulation. Point P1 is 1.5 mm from the bottom of the hole, and point P is 1.5 mm from the hole wall and 2.5 mm from the plane of the hole mouth.

As the drilling time increases, the heat generated in the cutting zone spreads to the inside of the workpiece through the workpiece’s own heat transfer. The maximum temperatures observed at point P1 were 145 °C and 131 °C for ordinary drilling and vibratory drilling, respectively, with a 9.6% reduction in the maximum temperature under vibratory drilling conditions. At the start of cutting, point P is far from the cutting zone, and the temperature rises more gently. At *t* = 4 s, i.e., at a drilling depth of 2 mm, the temperature starts to rise sharply. As the drilling depth continues to increase, point P moves away from the cutting zone, and the heat exchange environment at the hole wall is improved. As a result, the temperature starts to drop slowly, and the maximum temperature of ordinary drilling at point P is greater than that of vibratory drilling at point P. The results show that vibration drilling has improved the heat exchange conditions of the workpiece and has had a cooling effect.

Figure 8b shows the temperature change curve at the bottom of the hole and at the hole wall inside the workpiece, measured by a thermocouple with a drilling time of 50 s. At the initial moment, the temperature at each point is close to room temperature. As the drilling depth increases, the temperature at the hole wall starts to rise slowly, and the temperature change at the hole wall increases as it approaches the cutting zone. As the cutter teeth move away from the cutting area, less heat diffuses through the heat transfer to this point. Simultaneously, the cutting fluid comes into full contact with the machined hole surface and removes a large amount of heat, and the temperature at the hole wall begins to drop. The trend of temperature change at the workpiece hole wall is basically the same for both cutting methods. The highest temperatures measured at the hole wall were 63.4 °C and 59.8 °C for ordinary drilling and vibration drilling, respectively, with vibration drilling reducing the temperature by 5.7% compared to ordinary drilling. The temperature trend at the bottom of the hole differs between the two drilling methods. Due to the continuous cutting nature of ordinary drilling, heat spreads from the cutting zone to the inside of the workpiece from the start of the drilling. As the depth of drilling increases, the temperature rise accelerates until quasi-thermal equilibrium is reached at this point. The temperature rise at the bottom of the hole in the workpiece is gentler due to the intermittent cutting characteristics of vibration drilling. The highest temperatures measured at the bottom of the hole were 81.9 °C and 78.1 °C for ordinary drilling and vibration drilling, respectively, with vibration drilling being 4.6% lower than ordinary drilling.

From the simulation and experimental results, it can be concluded that the vibration drilling method can effectively improve the cutting heat exchange environment of the drilling system. At the same time, the temperature rise curves at the bottom of the hole and the hole wall inside the workpiece measured by the thermocouple are consistent with finite element simulation. However, due to the response time and measurement accuracy of the thermocouple sensor, as well as the influence of various complex factors in the actual drilling process, the temperature values obtained by the experiment and the finite element simulation have a slight error.

### 4.3. Wear Characteristics Analysis of Cutter Teeth Rake Face and Flank Face

**Figure 9:**
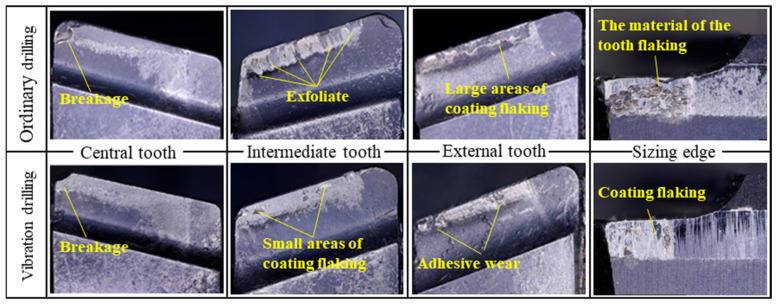
Wear pattern of the sizing edge and rake face of each tooth by different drilling methods.

During the drilling process of nickel-based alloys, the contact friction between the chip and the rake face of each cutter tooth leads to high cutting temperatures and easy crater wear on the rake face of the cutter teeth. The rake face of the cutter tooth wears, starting at the position with the highest cutting temperature as the center, and then gradually expanding backward and forward with increasing depth. When the crater develops to the point where the edge between the front of the rake face and the cutting edge is very narrow, the strength of the cutting edge is reduced, which can easily lead to cutting edge breakage [33]. Figure 9 shows the wear pattern of the rake face of each tooth and the sizing edge in the two cutting conditions of the nickel-based alloy. As seen from the Figure 9, the tip of the central tooth is broken in both conditions, and the simulation results show that the high-temperature area of the rake face of the central tooth is also concentrated in the tip position. This indicates that high temperatures and high cutting stresses are the main causes of breakage. Under ordinary drilling, the central tooth has obviously deeper crater wear. The rake faces of the intermediate and external teeth are more sharply worn. The sizing edge of the external teeth is severely worn. This has a serious impact on the stability of the drilling process and the quality of the hole. When vibration drilling, the rake face of each cutter tooth is evenly worn, and the wear of the intermediate and external teeth is significantly improved. This indicates that the vibration drilling method is effective in reducing the drilling temperature and, thus, improving tooth wear.

**Figure 10:**
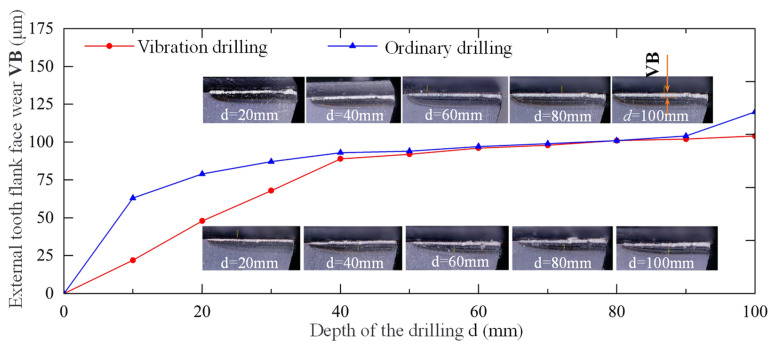
Variation of the wear width VB of the of the external teeth flank face with the same drilling depths between two drilling methods.

Tool wear can be divided into three stages, including initial wear stage, normal wear stage, and rapid wear stage. In the rapid wear stage, tool wear is accelerated, as well as noise, vibration, and so on. In the process of staggered tooth BTA drilling, the external tooth drilling speed is the largest and wear is faster, while the wear of the rake face, external tooth sizing edge, and guide block is not easy to measure and quantify. Therefore, in this paper, the mean wear width VB of the flank face of the external teeth was selected (as shown in Figure 10) to evaluate the degree of drill wear during the drilling of nickel-based alloy materials. Figure 10 shows the variation of the wear width VB of the external teeth flank face with the same drilling depths from two drilling methods. The drilling depth was 100 mm to study the wear law. In ordinary drilling, the tool teeth enter the normal wear stage after the drilling depth of 10 mm. During vibration drilling, when the drilling depth reaches 40 mm, the tool teeth enter the normal wear stage. The initial wear stage of vibration drilling is about three times longer than that of ordinary drilling, which prolongs the time for the tool to enter the normal wear stage. In the normal wear stage, the wear bandwidth of the external tooth flank face by two drilling methods is similar, about 0.1 mm. It can also be seen that when the drilling depth reaches 100 mm, the wear of the external teeth from ordinary drilling begins to enter the rapid wear stage, while the vibration drilling is still in normal wear stage. Therefore, vibration drilling can effectively hinder cutter teeth wear and improve the durability of the cutter teeth, meanwhile, nickel-based alloy materials have poor machinability and faster tool wear.

## 5. Conclusions

This paper investigated the variation of the temperature field of the cutter teeth throughout the cutting cycle during the drilling of nickel-based alloys via BTA vibration drilling and ordinary drilling. The variations in the temperature of the workpiece hole bottom and hole wall with time were analyzed. The wear patterns of each cutter tooth under the two drilling methods were compared, and the following conclusions were drawn.

(1) In the nickel-based alloy vibration drilling process, the drilling process is divided into a non-cutting phase and a cutting phase due to the cyclical disconnection of the drilling process. These two phases belong to the cooling time and the heating time, which can improve the cooling efficiency of the drills. Therefore, the cooling effect of the cutter teeth is obvious. Among them, the average temperatures of the central, intermediate and external teeth are reduced by 18.1%, 21.1%, and 17.8%, respectively, with a large temperature gradient of the cutter teeth and a small high-temperature area, which significantly improves the cutting heat exchange conditions of the drill.
(2) Under both conditions, the temperature of the workpiece changes with time in the same way. Simultaneously, the temperature at the bottom of the hole and at the wall of the hole in vibration drilling is lower than that in ordinary drilling, which means that vibration drilling has a good cooling effect on the workpiece.
(3) Cutting temperature is an important factor affecting tooth wear. The high-temperature area of the cutter teeth is also subject to severe wear. The vibration drilling method effectively prolongs the time of initial wear stage and normal wear of the cutter teeth and improve the durability of the cutter teeth.

## Figures and Tables

**Table 1:** The characteristic parameters of two materials [30,31].

Name of Parameter	Inconel 718	WC
Density/ρ(kg/m^3^)	8190	1500
Poisson’s ratio/μ	0.33	0.22
Specific Heat Capacity/c (J/kg·°C)	435	226
Thermal Conductivity/λ (W/m·°C)	14.7	44.6
Coefficient of Thermal Expansion/α (10^−6^/°C)	11.8	---

**Table 2:** Parameters of the J–C model for Inconel 718 material [32].

*A* (MPa)	*B* (MPa)	*C*	*n*	*m*
1241	622	0.0134	0.6522	1.3

**Table 3:** Drilling process parameters.

Drilling Method	Spindle Speed n (r/min)	Feed Rate *f_r_* (mm/r)	Amplitude *a* (mm)	Frequency Ratio ω*_f_*
Ordinary	600	0.05	---	---
Vibration	600	0.05	0.07	2.25

**Table 4:** Comparison of the temperature of each tooth between ordinary drilling and vibration drilling.

Drilling Methods	T_max_ (°C)			T_average_ (°C)		
	Central Tooth	Intermediate Tooth	External Tooth	Central Tooth	Intermediate Tooth	External Tooth
Ordinary drilling	570.8	605.7	685.0	563.1	589.4	668.0
Vibration drilling	522.6	578.5	638.3	461.1	464.9	549.2
Reduction	8.80%	4.47%	6.86%	18.1%	21.1%	17.8%

## Data Availability

The data presented in this study are available on request from the corresponding author.
